# Spatial solitons in an electrically driven graphene multilayer medium

**DOI:** 10.1038/s41598-022-15179-6

**Published:** 2022-06-29

**Authors:** Muzzamal Iqbal Shaukat, Montasir Qasymeh, Hichem Eleuch

**Affiliations:** 1 Electrical and Computer Engineering Department, Abu Dhabi University, 59911 Abu Dhabi, United Arab Emirates; 2 School of Natural Sciences, National University of Sciences and Technology, H-12, Islamabad, Pakistan; 3 Department of Applied Physics and Astronomy, University of Sharjah, Sharjah, United Arab Emirates; 4 Institute for Quantum Science and Engineering, Texas AM University, College Station, TX 77843 USA

**Keywords:** Optics and photonics, Other photonics, Solitons

## Abstract

We investigate the evolution of coupled optical solitons in a multilayer graphene medium. The considered graphene medium is subjected to microwave voltage biasing. The coupled two optical solitons emerge through the electrical (i.e., microwave voltage) perturbation of the effective permittivity of the graphene multilayer. We show that the coupled solitons are electrically adjustable by controlling the amplitude and frequency of the biasing microwave voltage. Importantly, this proposed regime of electrically controlled optical solitons offers a modality to generate entangled optical solitons and two-mode squeezed solitons. Furthermore, the hybrid interaction that includes both the driving microwave voltage and the optical solitons yields a platform to combine the two worlds of quantum photonics and quantum superconducting systems.

## Introduction

Over the past years, the scientific community has devoted significant efforts to studying the nonlinear Schrödinger equation while considering generalized multicomponent schemes^1–3^. In particular, solitons have attracted a lot of attention due to their significant propagating distances while shapes are preserved^4–9^. One of the most fundamental and ubiquitous phenomena in nonlinear systems is the Modulation Instability^10^, that occurs due to the interplay between dispersion and nonlinearity. Since the pioneering research of Manakov^11^ on vector solitons in coupled nonlinear Schrödinger equations, intense theoretical efforts have been devoted to the study of bright-dark^12,13^, dark-dark^14^ and bright-bright solitons^15,16^. We also note that soliton stability has been studied by investigating the externally (or parametric) driven damped nonlinear Schrodinger equation^17,18^. The coupled interactions play an important role to observe the collision dynamics in the system. Such models have attracted much attention in recent years, e.g., waveguides^19,20^ and optical fiber^21,22^.

On the other hand, an appealing property of graphene as a photonic medium lies in leveraging the possibility of tuning its conductivity by controlling the chemical doping or applying an external gate voltage^23,24^. For instance, recently, a novel approach of microwave-to-optical conversion has been introduced in graphene multilayer structure based on electrically perturbing the graphene conductivity^25–27^. Motivated by this approach, the present work aims to investigate the soliton formation of coupled nonlinear Schrödinger equations in a multilayer graphene structure. Enabled by the microwave voltage perturbation and by controlling the phase matching condition (e.g., by changing the period length of the multilayers), we show that coupled propagating solitons can be attained. As a result, we compute the dependence of soliton propagation on microwave parameters, i.e., amplitude $\nu$ and frequency $\omega _m$, and determine the significant propagation distances of coupled solitons by varying the medium’s period length and the intrinsic electron density.

In “Theoretical model” section, the problem statement is introduced, and the Manakov-type cross-coupled nonlinear Schrödinger equations (with self and cross phase modulation coefficients) are derived. Also, the numerical results are presented and explained. The conclusion is discussed in “Conclusion” section.

## Theoretical model

**Figure 1:**
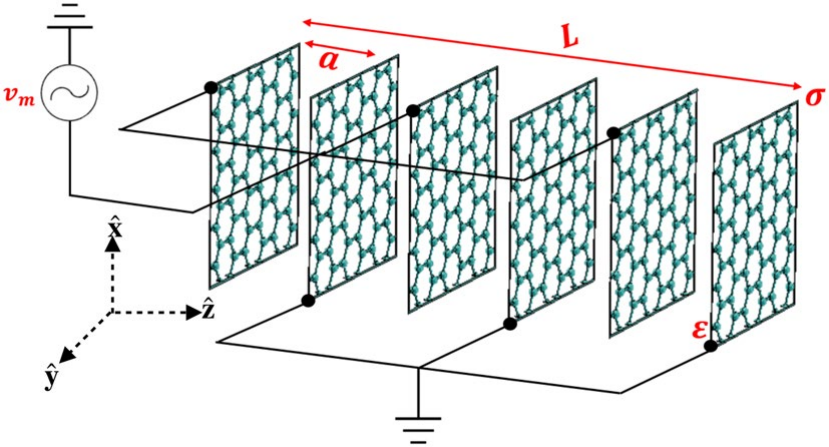
Schematic representation of the electrically driven graphene multilayer medium.

The studied medium is a multilayer graphene structure (closely related to Ref.^25–27^), shown in Fig. 1. The graphene layers are electrically connected in an interdigital model forming a parallel plate capacitors configuration. The medium is of a length *L*, the microwave signal biasing is with frequency $\omega _m$, and the two optical fields have distinct frequencies $\omega _1$ and $\omega _2$. Importantly, the electro-optic interaction is enabled by satisfying the condition $\omega _1-\omega _2=\omega _m$. We note that having this condition fulfilled allows the perturbed effective permittivity to couple the optical solitons with the driving microwave field (see^25–27^ for more details ). It then follows that for proper medium parameters, the electrically controlled optical solitons are attained (as demonstrated in the remaining part of this work). To illustrate the nonlinear soliton formation in a multilayer graphene structure, we employ the generic method formulated in term of Helmholtz’s equation^5,28,29^, given by:

1 $$\begin{aligned} \nabla ^2 E_j-\frac{1}{c^2}\frac{\partial ^2\big ( \epsilon _{eff}E_j\big )}{\partial t^2}= \frac{1}{\epsilon _{0}c^2}\frac{\partial ^2P_{nl}}{\partial t^2}, \end{aligned}$$

where $E_j(x,z,t)$ is the electric field, $j\in {1,2}$, $\epsilon _0$ is the free space permittivity, $\epsilon$ denotes the filling material permittivity, and *c* remarks the speed of light. Here, $P_{nl}(x,z,t)=\epsilon _{nl} E_j(x,z,t)$ denotes the nonlinear polarization density, and $\epsilon _{nl}=\epsilon _{0}\chi ^{(3)}|E^i_{nl}(x,z,t)|^2$ is the nonlinear graphene permittivity. The optical field in the multilayer graphene is defined as:

2 $$\begin{aligned} E_j(x,z,t)=\sum _{j=1}^2 A_j(z,x) e^{-i(\omega _j t -\beta _j z)} {\hat{y}} +c.c, \end{aligned}$$

where $A_j(z,x)$ indicates the slow varying amplitude, and $\omega _j$ represents the distinct frequencies of the optical field. The optical propagation constant $\beta _j$ is obtained from the dispersion relation^30^:

3 $$\begin{aligned} \mathrm{Cos}(a \beta _j)=\mathrm{Cos}\left( a\sqrt{\epsilon }\frac{\omega _j}{c}\right) -i\frac{\eta _0}{2\sqrt{\epsilon }}\mathrm{Sin}\left( a\sqrt{\epsilon }\frac{\omega _j}{c}\right) \sigma ^j_s, \end{aligned}$$

where *a* is the period length, and $\eta _0=377\Omega$ is the free space impedance. The graphene conductivity $\sigma ^j_s$ is given by^31^:

4 $$\begin{aligned} \sigma ^j_s= & {} \frac{i e^2}{4\pi \hbar }\mathrm{ln}\left\{ \frac{4\pi \mu _c-\hbar (\omega _j+i2\pi \Gamma )}{4\pi \mu _c+\hbar (\omega _j+i2\pi \Gamma )} \right\} \nonumber \\&+\frac{i 2 e^2 k_B T}{ \hbar ^2(\omega _j+i2\pi \Gamma )} \left\{ \frac{\mu _c }{k_B T}+2\mathrm{ln\left\{ 1+e^{-\frac{\mu _c}{k_B T}} \right\} } \right\} , \end{aligned}$$

where *e* is the elementary electric charge, $\hbar$ represents the Planck constant, *T* is the temperature and $\Gamma =1/\tau$ is the loss factor in graphene. Here, $\mu _c=\hbar v_F\sqrt{\pi n_0+2C_T V_m/e}$ denotes the chemical potential of graphene with the driving microwave voltage $V_m=\nu e^{-i\omega _m t}$+c.c, and $C_T=4(N-1)\epsilon \epsilon _0/a$ is the electrical capacitance of the graphene layers. By employing the approximation $\sqrt{1+A}\simeq 1+A/2$, for $A\ll 1$, the chemical potential $\mu _c$ can be expanded to the first order, yielding $\mu _c=\mu '_c+\nu \mu ''_c e^{-i\omega _m t}+c.c.$ Consequently, the graphene’s conductivity approximated to the first order is given by^25–27^:

5 $$\begin{aligned} \sigma _s=\sigma '_s+\nu \sigma ''_s e^{-i\omega _m t}+c.c. \end{aligned}$$

Here, $$\nu \sigma ''_s \ll \sigma '_s$$, $$\sigma '^j_s=\sigma ^j_s$$, and

6 $$\begin{aligned} \sigma ''^j_s= & {} \frac{i e^2}{2\pi ^2}{\left\{ \frac{(\omega _j+i2\pi \Gamma )}{(4\pi \mu '_c)^2+\hbar ^2(\omega _j+i2\pi \Gamma )^2} \right\} }\mu ''_c\nonumber \\&+\frac{i 2 e^2 k_B T}{ \hbar ^2(\omega _j+i2\pi \Gamma )} \mathrm{tanh}\left( \frac{\mu '_c}{2K_B T}\right) \frac{\mu ''_c}{K_B T}. \end{aligned}$$

The unperturbed graphene chemical potential is given by $\mu '_c=\hbar v_F \sqrt{\pi n_0}$, and the perturbation of graphene chemical potential is given by $\mu ''_c=\hbar v_F c_T/(e\sqrt{\pi n_0})$. By considering $C_T \nu \ll e\pi n_o$, the effective permittivity $\epsilon _{eff}$ and the propagation coefficient $\beta$ can be decomposed, similar to Eq. (5), as:

7 $$\begin{aligned} \epsilon _{eff}= & {} \epsilon _{eff}'+\nu \epsilon _{eff}'' e^{-i\omega _m t}+c.c., \nonumber \\ \beta _j= & {} \beta '_j+\nu \beta ''_j e^{-i\omega _m t}+c.c, \end{aligned}$$

where $$\epsilon '^j_{eff}=\beta '_j/k_j$$, $$\epsilon ''^j_{eff}=2\beta '_j\beta ''_j/k^2_j$$ and

8 $$\begin{aligned} \beta ''^j=i\frac{\eta _0}{2 a \sqrt{\epsilon }}\frac{\mathrm{Sin}\left( \frac{a \omega _j \sqrt{\epsilon }}{c} \right) }{\mathrm{Sin}\left( a \beta ' \right) } \sigma ''^j_s, \end{aligned}$$

and $\beta '_j$ can be obtained from Eq. (3). It is relevant to note here that the present work aims to provide a rigorous theoretical foundation to illustrate the feasibility of electrically controlling optical solitons utilizing a multilayer graphene system. Furthermore, the presented modality includes hybrid interaction involving microwave and optical fields. Hence, offering a promising potential to integrate the two domains of quantum photonics and quantum superconducting systems. Additionally, achieving re-configurable devices based on the illustrated feature of electrically adjustable coupled solitons is another promising property.

By employing Eqs. (2) and (7) with the slowly varying envelope approximation $\partial ^2 A_j/\partial z^2 \ll 2 i \beta _j \partial A_j /\partial z$ (i.e., slow envelope variations with negligibly small second z-derivative), one can derive a simplified coupled equations for amplitude $A_j$ as follows:

9 $$\begin{aligned} & \frac{{\partial ^{2} A_{1} }}{{\partial x^{2} }} + 2i\beta _{1} \frac{{\partial A_{1} }}{{\partial z}} + 2\beta ^{\prime}_{1} \beta ^{\prime\prime}_{1} \left( {1 + \frac{{\omega _{m} }}{{\omega _{1} }}} \right)^{2} \nu A_{1} \\ & + k_{1}^{2} \chi ^{{(3)}} \left( {|A_{1} |^{2} + \kappa |A_{2} |^{2} } \right)A_{1} = 0, \\ & \frac{{\partial ^{2} A_{2} }}{{\partial x^{2} }} + 2i\beta _{2} \frac{{\partial A_{2} }}{{\partial z}} + 2\beta ^{\prime}_{2} \beta ^{\prime\prime}_{2} \left( {1 + \frac{{\omega _{m} }}{{\omega _{2} }}} \right)^{2} \nu ^{*} A_{2} \\ & + k_{2}^{2} \chi ^{{(3)}} \left( {\kappa |A_{1} |^{2} + |A_{2} |^{2} } \right)A_{2} = 0, \\ \end{aligned}$$

where $\kappa$ denotes the coupling parameter, $\chi ^{(3)}=W\{ 1+i\nu \left( 1+\omega _m/\omega _1\right) ^2 \mu _{c}''/\mu _c'\}/(\mu _c')$ is the graphene third-order nonlinear susceptibility (which is a Kerr-like type), and $W=3e^4v_F^2/(16\pi ^2\hbar ^2\omega _1^4 d)$^32–34^. The equation in (9) are obtained under the conditions $\nu \mu ''_c\ll \mu '_c$ and $\mathrm{Im}[\beta ''_j] \ll \mathrm{Re}[\beta ''_j]$^34^.

**Figure 2:**
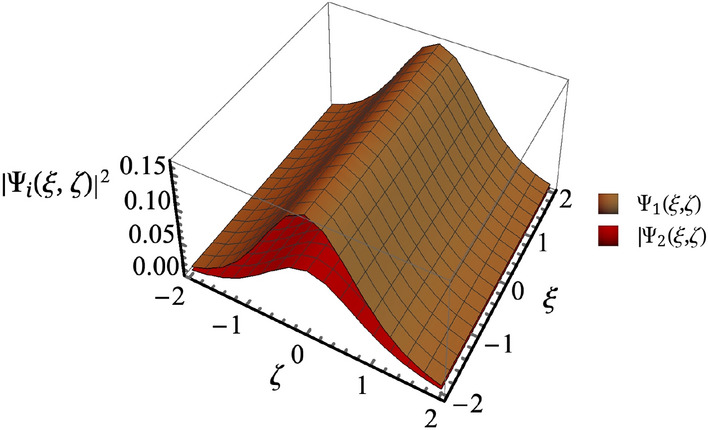
Normalized solitons spatial profile of Eq. (12). Here, $\omega _1/2\pi =195.6 \; \mathrm{THz}$ and $\omega _2/2\pi =185.6 \; \mathrm{THz}$.

For simplification, an auxiliary function $\Psi _{i}= \Lambda _0 k_1\sqrt{\chi ^{(3)}} A_i e^{- i \alpha _i z}/\sqrt{2}$ is defined with the normalization parameters $\xi =z/ \beta _1 \Lambda _0^2$ and $\zeta =x /\Lambda _0$, where $\alpha _i=\beta ''_i (1+\omega _m/\omega _i)^2 \nu _i$ and $\Lambda _0$ denotes the beam width. In what follows, Eq. (9) simplifies to:

10 $$\begin{aligned}i \frac{\partial \Psi _1}{\partial \xi } +\frac{1}{2}\frac{\partial ^2 \Psi _1}{\partial \zeta ^2} + \left( |\Psi _1|^2+2|\Psi _2|^2 \right) \Psi _1=0, \end{aligned}$$

11 $$\begin{aligned}i \delta \frac{\partial \Psi _2}{\partial \xi } +\frac{1}{2}\frac{\partial ^2 \Psi _2}{\partial \zeta ^2} +\alpha \left( 2|\Psi _1|^2+|\Psi _2|^2 \right) \Psi _2=0, \end{aligned}$$

with $\delta =\beta _2/\beta _1$ and $\alpha = k_2^2/k_1^2$. First, before analyzing the solution of Eqs. (10) and (11), the case of two separated waves can be considered by neglecting the cross coupling terms, which leads to a well known bright soliton solution $\Psi =\mathrm{Sech}(\zeta )e^{i\xi /2}$^35^. Qasymeh in^30^ has proposed a novel terahertz amplification technique utilizing a similar graphene layered medium with two optical waves implemented. He has considered the evolution of the parameter $\beta$ versus the optical frequency for different period lengths (see Fig. (2) of Ref.^30^) and depicted that the optical frequencies $\omega _1$ and $\omega _2$ can be chosen symmetrically above and below the medium resonance to satisfy the phase matching condition $\beta _1-\beta _2=0$. Thanks to this property of the multilayer graphene system, we employ the same approach to simplify Eqs. (10) and (11) by considering $\delta =\beta _2/\beta _1=1$. Furthermore, Raul in^36^ has investigated solitons in an optical Kerr medium. He considered the case of superimposing two solitons that co-propagate while accommodating self-phase and cross-phase modulation terms. By comparing Eqs. (10) and (11) of the present investigation with Eq. (5) of Ref.^36^, we can see that the current scheme possesses the novel effect of microwave voltage controlling property while the parameter $\alpha$ varies as $0.5<\alpha <2$ ($\alpha \ne 1$). Consequently, by following the procedure outlined in Ref.^36^, we have depicted the solution of Eq. (10) by direct substitution,

12 $$\begin{aligned} \Psi _i=U_i \mathrm{Sech}\left( \zeta \right) e^{i\xi /2}, \end{aligned}$$

where the amplitudes are given by:

13 $$\begin{aligned} U_{1}=\sqrt{\frac{2\omega ^2_{1}-\omega ^2_{2}}{3\omega _2^2}}, U_{2}=\sqrt{\frac{2\omega ^2_{2}-\omega ^2_{1}}{3\omega _2^2}}. \end{aligned}$$

Figure 2 depicts the soliton formation for the normalized solution (Eq. (12)) of coupled equation (10), which can also be numerically verified using Eq. (9). The soliton power can be described by $P_{1,2}=4U_{1,2}/k_{1,2}^2 \Lambda _0 \chi$^37^. It is worth mentioning here that the spatial soliton in Eq. (12) can be compared to the temporal soliton in Eq. (5.11) of Ref.^38^ by having proper transformation (e.g. $U_i \rightarrow 1/\sqrt{3}$, $\zeta \rightarrow X$ and $\xi /2 \rightarrow T$). It then follows that we can borrow the procedure outlined in the Ref.^38^ and characterize the soliton stability. On the other hand, the spatial solitons considered in the current work always satisfy the condition $\omega _1-\omega _2=\omega _m$ ( which is needed to enable the interaction of the optical and microwave fields). Hence, it not possible to have $\alpha =k_2^2/k_1^2=1$ or $\omega _1=\omega _2$. Interesting research directions include solitons interactions with different external potential configurations and other forms of generalized coupled NLSE. We plan to address these aspects in our future work.

**Figure 3:**
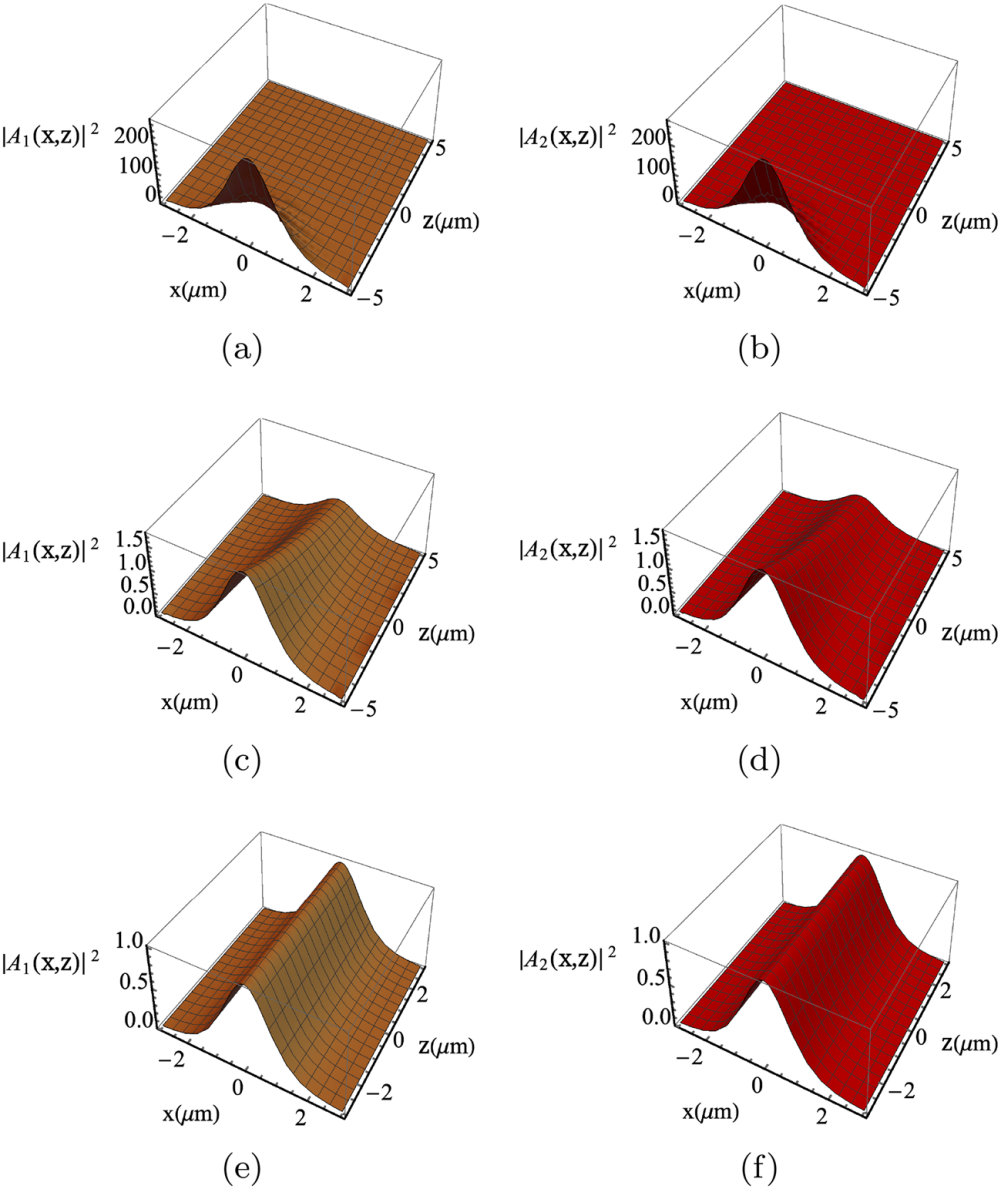
Solitons profile considering different microwave voltages amplitude (i.e., $\nu$). The solitons profile are displayed for $\nu =1\;\text{mV}$ (**a**,**b**), $\nu =0.1 \; \text{mV}$ (**c**,**d**) and $\nu =1 \;\upmu \text{V}$ (**e**,**f**), respectively. The solitons profiles are preserved for significant propagation distance $\sim 5 \; {\upmu \text{m}}$ in (**e**,**f**), while distorted even for a relatively short propagation distance $\sim 1{-}2\; {\upmu \text{m}}$ in (**a**,**c** or **b**,**d**). The other parameters are $\omega _1=2\pi \times 193\; \mathrm{THz}$, $\omega _2=2\pi \times 192.88 \; \mathrm{THz}$ and $\omega _m=2\pi \times 120 \; \mathrm{GHz}$, the period length $a=1 \; \; {\upmu \text{m}}$, the number of graphene layers $N=10$, the intrinsic electron density $n_0=2\times 10^{14}\;\text{m}^{-2}$, the susceptibility $\chi =2.095 \;f$, loss factor $\Gamma =1\;\mathrm{THz}$ and the permittivity $\epsilon =(3.5)^2$.

Although we have analytical results with the procedure outlined in Ref.^36^, we investigate Eq. (9) numerically to observe the role of the microwave voltage parameters on the optical soliton dynamics. In the panels (a,c) and (b,d) in Fig. 3, the coupled solitons are subjected to microwave biasing with amplitudes 1 mV and 0.1  mV, respectively. It can be seen the the soliton profiles are severely distorted even for a very short propagation distance ($1 \; {\upmu \text{m}}$ and $2 \; {\upmu \text{m}}$, respectively). On the other hand, in the panels (e,f) of the same figure, the microwave voltage amplitude is altered to be $\nu =1 \; {\upmu \text{V}}$. As a result, the coupled solitons are shown to be stable and preserve their shapes even after large propagation distance($\sim 5 \; {\upmu \text{m}}$). This is very interesting observation illustrating the optical control over the propagating optical solitons.

**Figure 4:**
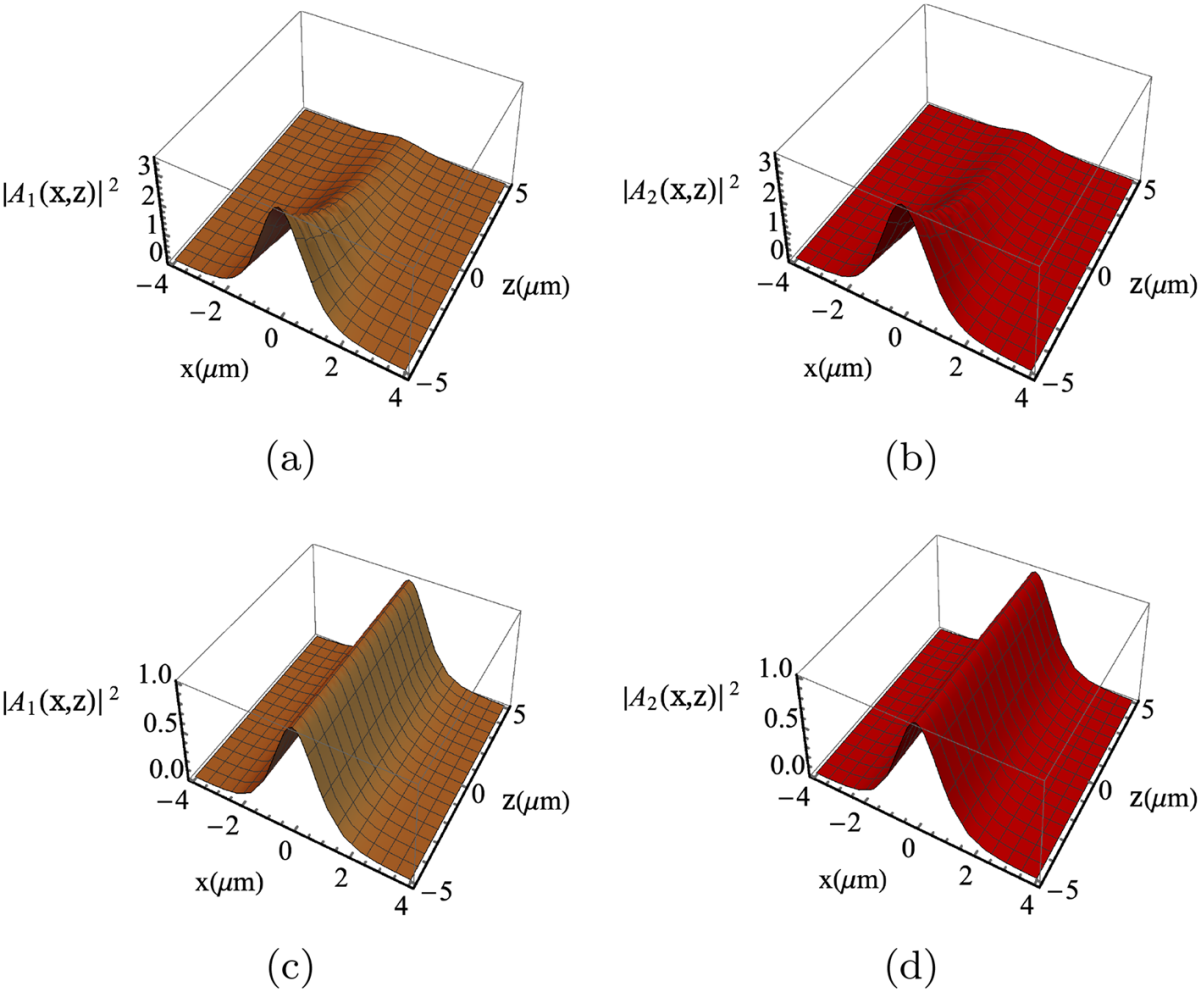
Solitons profile considering the microwave field $\omega _m/2\pi =90 \; \mathrm{GHz}$. In (**a**,**b**) the solitons profile are severely distorted. In (**c**,**d**), the solitons profile can be re-establishes for significant propagation distances by altering the period length to be $a= 10\; {\upmu \text{m}}$ ($N=100$). Here, $\omega _1=2\pi \times 193 \; \mathrm{THz}$, $\omega _2=2\pi \times 192.91 \; \mathrm{THz}$ and other parameters are same as in Fig. 3.

**Figure 5:**
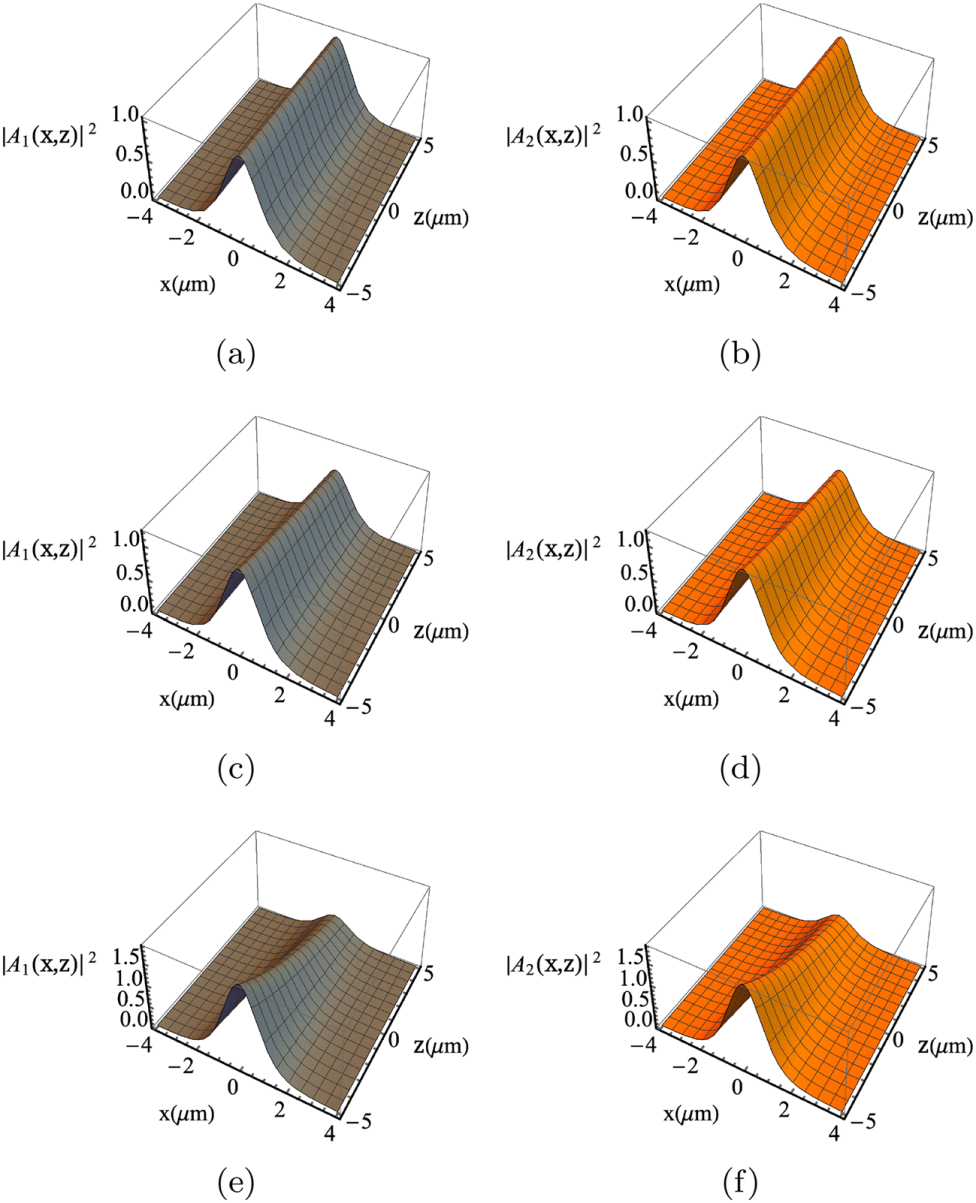
Solitons profile for different number of graphene layers. The number of graphene layers are $N=5$ for (**a**,**b**), $\hbox {N}=30$ for (**c**,**d**), and N = 100 for (**e**,**f**). Here, $\omega _1=2\pi \times 193.09 \; \mathrm{THz}$, $\omega _2=2\pi \times 193 \; \mathrm{THz}$, the period length $a=1 \; {\upmu \text{m}}$, the susceptibility $\chi =2.095\; f$, the intrinsic electron density $n_0=2\times 10^{14}\;\text{m}^{-2}$, $\nu =1 \; {\upmu \text{V}}$, the temperature $T=30\;\mathrm{K}$, the loss factor $\Gamma =1\;\mathrm{THz}$ and the permittivity $\epsilon =(3.5)^2$.

**Figure 6:**
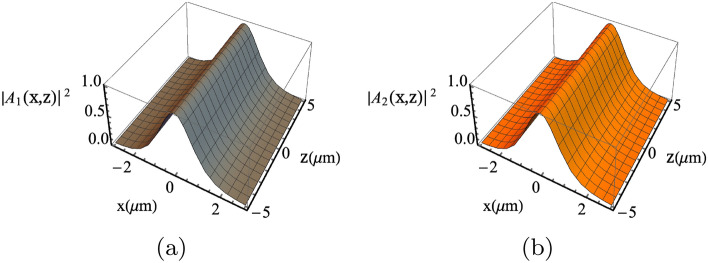
Solitons profile considering period length $a=10 \; {\upmu \text{m}}$ and $N=100$. Other parameters are the same as in Fig. 5.

Figure 4 displays the effect of varying the microwave frequency $\omega _m$ on the soliton profile formation (while the phase matching condition is always fulfilled). It is depicted that changing the microwave frequency from its optimized value results in reducing the soliton propagation distance, as shown in Fig. 4a,b. Nonetheless, thanks to the geometry of the graphene layered structure, the soliton can be reestablished for large propagating distances by properly adjusting the period length of the graphene multilayers, as demonstrated in Fig. 4c,d. The variation of the soliton structure with the number of the graphene layers is presented in Fig. 5. Basically, the number of graphene layers determines the propagation distance at which the amplitude of the coupled solitons decreases and the profiles get distorted for a large number of graphene layers (see Fig. 5e,f). However, the soliton propagation can be reestablished by modifying the period length (see Fig. 6).

Finally, we give here a brief discussion on some crucial differences between our scheme and others. In the literature, the self-phase modulated (SPM) spatial solitons are reported in graphene based structures^39–42^. The present investigation (scheme) describes the novel configuration of biased microwave voltage to propose the self and cross-phase modulated spatial solitons. Furthermore, the phase matching condition $\beta _1=\beta _2$^34^ allows to obtain the Manakov type equation. As a result, we get a functional control of soliton propagation through adjusting the biasing microwave parameters. It is worth noting that the two coupled solitons have the potential to provide novel functionalities in both conventional and quantum technology. One possible domain is the error corrections in a classical communication system. Likewise, entangled and squeezed two-mode solitons can be established based on the proposed coupled solitons, promising future quantum advances and applications.

## Conclusion

In conclusion, we have investigated a novel approach of coupled soliton formation in multilayer graphene structure with optical and microwave field parameters. We have taken the self and cross phase-modulated coefficients into account and simplified the governing coupled nonlinear Schrödinger equations by designing the layers’ periodicity length to satisfy the phase-matching condition. Furthermore, the propagation of these coupled solitons for a large number of graphene layers can be attained by varying the microwave biasing and the intrinsic electron density. The coupled solitons appear as a result of the proposed microwave-optical fields interaction. This interaction is accomplished through electrically perturbing the graphene conductivity. It is also shown that these coupled solitons possess novel electrically enabling features by which the solitons can be switched on and off. This electronic control feature (which can be seized to attain tunable functionality) is only possible by accommodating two coupled solitons (since the interaction is enabled by setting the frequency difference between the two solitons equal the driving microwave frequency). Potential future applications of the proposed scheme have been highlighted and discussed.

## Data Availability

The datasets generated during and/or analysed during the current study are available from the corresponding author on reasonable request.
